# Two-Dimensional Triblock Peptide Assemblies for the Stabilization of Pickering Emulsions with pH Responsiveness

**DOI:** 10.1021/acsami.2c17558

**Published:** 2022-11-15

**Authors:** Zhiwei Huang, Eleonora Calicchia, Izabela Jurewicz, Edgar Muñoz, Rosa Garriga, Giuseppe Portale, Brendan J. Howlin, Joseph L. Keddie

**Affiliations:** † Department of Physics, Faculty of Engineering and Physical Sciences, University of Surrey, GuildfordGU2 7XH, U.K.; ‡ Groningen Research Institute of Pharmacy, University of Groningen, A. Deusinglaan 1, Groningen9713 AV, The Netherlands; § Zernike Institute for Advanced Materials, Faculty of Mathematics and Natural Sciences, University of Groningen, Nijenborgh 4, Groningen9747AG, The Netherlands; ∥ Instituto de Carboquímica ICB-CSIC, Miguel Luesma Castán 4, 50018Zaragoza, Spain; ⊥ Departamento de Química Física, Universidad de Zaragoza, 50009Zaragoza, Spain; # Department of Chemistry, Faculty of Engineering and Physical Sciences, University of Surrey, Guildford, Surrey GU2 7XH, U.K.

**Keywords:** two-dimensional nanomaterials, peptide amphiphiles, colloidal tectonics, self-assembly, Pickering emulsion, pH responsiveness, tectomer

## Abstract

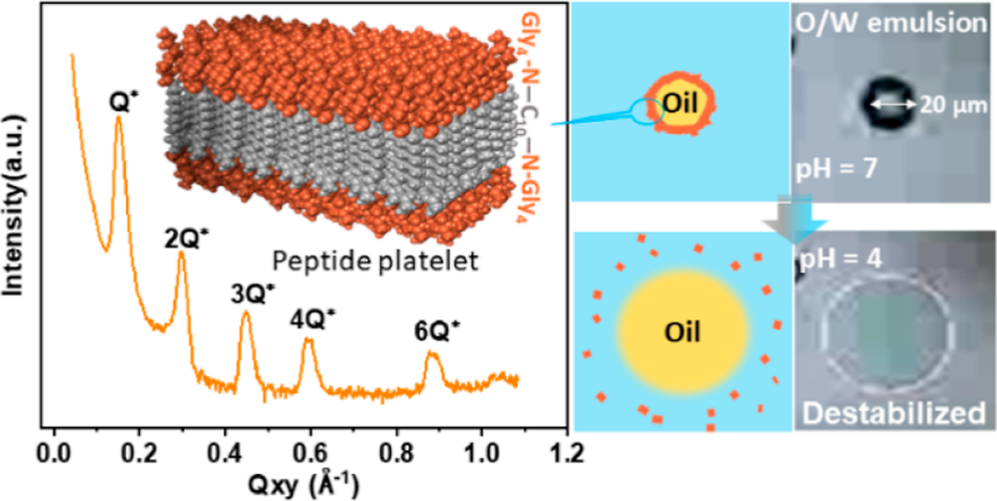

A variety of two-dimensional (2D) nanomaterials, including graphene oxide and clays, are known to stabilize Pickering emulsions to fabricate structures for functions in sensors, catalysts, and encapsulation. We introduce here a novel Pickering emulsion using self-assembled amphiphilic triblock oligoglycine as the emulsifier. Peptide amphiphiles are more responsive to environmental changes (e.g., pH, temperature, and ionic strength) than inorganic 2D materials, which have a chemically rigid, in-plane structure. Noncovalent forces between the peptide molecules change with the environment, thereby imparting responsiveness. We provide new evidence that the biantennary oligoglycine, Gly_4_–NH–C_10_H_20_–NH–Gly_4_, self-assembles into 2D platelet structures, denoted as tectomers, in solution at a neutral buffered pH using small-angle X-ray scattering and molecular dynamics simulations. The molecules are stacked in the platelets with a linear conformation, rather than in a U-shape. We discovered that the lamellar oligoglycine platelets adsorbed at an oil/water interface and stabilized oil-in-water emulsions. This is the first report of 2D oligoglycine platelets being used as a Pickering stabilizer. The emulsions showed a strong pH response in an acidic environment. Thus, upon reducing the pH, the protonation of the terminal amino groups of the oligoglycine induced disassembly of the lamellar structure due to repulsive electrostatic forces, leading to emulsion destabilization. To demonstrate the application of the material, we show that a model active ingredient, β-carotene, in the oil is released upon decreasing the pH. Interestingly, in pH 9 buffer, the morphology of the oil droplets evolved over time, as the oligoglycine stabilizer created progressively a thicker interfacial layer. This demonstration opens a new route to use self-assembled synthetic peptide amphiphiles to stabilize Pickering emulsions, which can be significant for biomedical and pharmaceutical applications.

## 1 Introduction

### 1.1 2D Nanomaterials and Their Use in Pickering Emulsions

Two-dimensional (2D) nanomaterials possess sheet or plate structures with lateral sizes greater than 100 nm but thicknesses of a few nanometers or less.^1^ They typically have strong in-plane bonding and weak out-of-plane interactions. These materials have been proven to have unique chemical reactivity and physical properties, promising potential applications in electronics, catalysis, energy storage and generation, sensing, separation, and related fields.^1,2^

Recently, a new range of applications using 2D nanomaterials as emulsion stabilizers has been established.^3^ Many 2D nanomaterials have been developed to stabilize Pickering emulsions, including graphene oxide (GO) nanosheets,^4^ clay platelets,^5^ and 2D polymer sheets.^6^ Compared to other types of stabilizers, such as traditional amphiphilic surfactants^7^ or Pickering nanoparticles,^8^ 2D nanomaterials are expected to lay flat at fluid–fluid interfaces and present a large surface area. It has been theoretically and experimentally shown that a very low concentration of amphiphilic nanosheets can provide good stabilization for a large area of liquid–liquid interfaces. For example, GO has a unique 2D sheet structure of carboxylic acid at the edges and phenol hydroxyl and epoxide groups mainly on the basal plane. Exploiting this chemical structure, Kim and co-workers^9^ were the first to produce stable GO-stabilized Pickering emulsions with a low GO concentration. They showed that GO can successfully disperse insoluble solids in aqueous media in a similar way as molecular surfactants. Later on, through analyzing the free energy of ultrathin platelike 2D materials at the oil/water interface, Creighton and co-workers^10^ developed a thermodynamic model to predict the behavior of 2D materials at liquid–liquid interfaces by studying the influence of material thickness, surface chemistry, and the extent of van der Waals transparency. The authors used GO as an example and demonstrated its unique molecular barrier properties when in multilayer tiling.

2D nanomaterials can stabilize Pickering emulsions and impart unique properties. The assembly of 2D nanomaterials at an oil/water interface can be used to construct advanced microstructures. For example, taking the advantage of the affinity of H_2_ to molybdenum disulfide (MoS_2_) nanosheets, Park et al.^11^ fabricated architectures from platinum (Pt)-decorated MoS_2_-stabilized Pickering emulsions for rapid hydrogen sensing with a fast response and recovery speed. Macroporous GO-polymer hybrids with potential applications in energy management were produced by Zheng and co-workers,^12^ where they used water-in-oil emulsions as a template. They fabricated a 3D carbon framework after polymerization and calcination of the GO-stabilized emulsion. Using 2D-catalyst-material-stabilized Pickering emulsions as a microreactor has also attracted great attention. Shan et al.^13^ prepared highly thermodynamically stable emulsions using layered double hydroxide (LDH) nanosheets and carbon nanotubes. The emulsion droplets with a small size provided large and stable interfacial areas for reactions, leading to high catalytic performance for the selective oxidation of benzyl alcohol. Furthermore, Pickering emulsions stabilized by 2D nanomaterials allow the controlled deposition of interfacial films on a substrate without disruption by the “coffee-ring effect” during drying, therefore making it possible to cast a uniform film from Pickering emulsion inks.^14^ Exploiting this benefit, Ogilvie et al. fabricated conductive networks from GO or MoS_2_ nanosheets (at volume fractions as low as 10^–5^)-stabilized water-in-oil Pickering emulsions.^15^ Pickering emulsions stabilized by 2D polymer nanosheets have also been explored for the delivery of oil-soluble pharmaceuticals.^6^ Clearly, increasing the diversity of 2D materials used to prepare Pickering emulsions will open new applications.

### 1.2 2D Peptide Assemblies and Their Use in Pickering Emulsions

In this research, we introduce 2D peptide assemblies as a new class of Pickering emulsion stabilizers. These assemblies are made from triblock peptide amphiphiles consisting of hydrophobic components and covalently conjugated hydrophilic peptides. Peptide amphiphiles can self-assemble into a variety of supramolecular nanostructures, including micelles,^16^ ribbons,^17^ nanofibers,^18^ nanotubes,^19^ and lamellar sheets,^20^ because of intermolecular noncovalent forces, such as hydrogen bonds, hydrophobic interactions, electrostatic interactions, aromatic interactions (π–π stacking), and nonspecific van der Waals attractions.^21^

Peptide assemblies are not the only type of material that enables the formation of hierarchical systems with predictable, versatile, and switchable properties. Recently, Leclercq introduced the new concept of “colloidal tectonics”, which is used to describe the interactions between molecular species to obtain a variety of self-assembled systems called (supra)colloids.^22^ However, compared to other tectons (the molecular building blocks for the (supra)colloidal structures), such as polyoxometallates,^23^ cyclodextrins,^24^ and polymers,^25^ peptide amphiphiles have attracted a huge amount of attention in recent decades as they combine structural characteristics of amphiphilic surfactants with the bioactive functionality of peptides.^26^ Furthermore, peptide nanostructures are generally biocompatible and biodegradable. At the same time, a high density of biological signals can be displayed on their surface, suggesting a significant promise for biomedical applications.^27^

Unlike 2D materials, such as GO and MoS_2_, which have a chemically rigid, in-plane structure, 2D self-assembled peptide amphiphiles are more responsive to environmental changes (e.g., pH, temperature, and ionic strength). This is because the noncovalent forces between the peptide’s molecules are changed with the environment, allowing the self-assembly and corresponding morphology to be adjusted.^28,29^ This attractive feature also provides an alternative route for the design and fabrication of functional supramolecular assemblies and materials.^30^ This prior work has inspired us to propose the use of peptide amphiphiles to produce stimuli-responsive Pickering emulsions.

Synthetic amphiphilic diblock polypeptide molecules have been used as conventional emulsifiers. For example, Hanson et al. produced stable water/oil/water double emulsion by using a single-component copolypeptide (K_*x*_(rac-L)_*y*_) as the surfactant.^31^ Recently, it was reported that peptide amphiphiles ((Ala)_9_–Arg) stabilize emulsions through the absorption of fiber networks at the oil/water interface.^32^ There are also recent reports that by changing the peptide sequences, aromatic tripeptide emulsifiers are able to stabilize emulsions either by forming fiber networks or acting as conventional surfactants.^33^ Recent computer simulations^34^ have discovered that a few particular amphiphilic tetrapeptides can reduce the interfacial tension of the oil/water interface and therefore are promising candidates for emulsifiers. Of these, a tetrapeptide that contained glycine units was particularly effective. In these diblock amphiphiles, the peptides do not assemble into larger structures, such as ribbons, but are expected to adsorb as individual molecules at the oil/water interface.

### 1.3 Self-Assembly of pH-Responsive Two-Tailed Oligoglycine

Biantennary oligoglycine (Gly_4_–NH–C_10_H_20_–NH–Gly_4_, or C_10_(NGly_4_)_2_), which consists of a middle alkyl chain and four glycine amino acid residues (tetrapeptides) on both sides is our focus here. Upon spontaneous self-assembly, C_10_(NGly_4_)_2_ forms “polyglycine II” structures, in which peptide helices 3_1_ (ϕ = −76.9°, ψ = 145.3°) form a network of hydrogen bonds with six neighboring chains, extending in two dimensions.^35−37^ This type of 2D assembly is also called a “tectomer” in the literature.^38,39^ The “two-tailed” (2T) oligoglycine creates assemblies in the aqueous phase, which we call 2T tectomers, or simply 2T for convenience.

There are only a few studies of 2T self-assembly on mica surfaces from aqueous solution. Tsygankova et al.^40^ inferred that 2T might form mono- or bilayers by studying 2T after its adsorption on a mica surface using atomic force microscopy (AFM). For the primary monolayer, a “2 + 0” conformation is presumed, where two peptide antennae are adsorbed on the mica surface because of electrostatic interactions between the positively charged terminated amino group and the negatively charged mica surface, while the hydrophobic linker is outside facing the water phase. Simultaneously, a second layer can be formed with an opposite peptide orientation on the hydrophobic surface of the resultant layer probably because of hydrophobic interactions between −(CH_2_)_10_– moieties. Experimental observations of adsorbed layers agree with the computer simulations performed by Gus’kova and co-workers.^41^

The pH responsiveness of 2T oligoglycine assemblies has been investigated in depth by some of the authors.^42,43^ We found that in acidic solutions (pH 3.0), 2T tectomers disassemble because of the strong electrostatic repulsion between protonated terminal amino groups. However, when the pH of the solution was increased to 7.4, a massive aggregation was triggered because of the deprotonation of the amino groups. Plate-like structures were formed and precipitated out of the solution. Moreover, we explored 2T as an effective pH-responsive nanocarrier, with potential for attractive biosensing and therapeutic applications. In another work,^43,44^ we reported that 2T assemblies formed on carboxylated multiwalled carbon nanotubes and GO fibers to fabricate free-standing, conducting composites or pH-switchable bioadhesive coatings.

Herein, we will demonstrate, for the first time, novel Pickering emulsions stabilized by 2D peptide triblock amphiphiles—instead of carbon-based or other related crystalline nanomaterials—and show pH sensitivity in the emulsion. We explore the conditions that enabled 2T to stabilize emulsions and then investigated the self-assembly mechanism. Using small-angle X-ray scattering (SAXS), we present the structures of the 2T assembly in solution and in films formed at the oil/water interface. We also explore the effect of the protonation of the terminal amino group of C_10_(NGly_4_)_2_ to impart pH responsiveness to the emulsion. We use molecular dynamics (MD) simulations to investigate the mechanism of the tectomer self-assembly and to deepen our understanding. We also discuss their potential applications.

## 2 Materials and Methods

### 2.1 Materials

C_10_(NGly_4_)_2_ or Gly_4_–NH–C_10_H_20_–NH–Gly_4_ (purity >95%) was purchased from PlasmaChem GmbH (Berlin, Germany) and used as received. Pure sunflower oil (Flora, Princes Ltd, UK) was used as the oil phase (without any purification) to prepare emulsions. Buffer tablets pH 4.0 (phthalate), pH 7.0 (phosphate), and pH 9.2 (borate) were all obtained from Fisher Scientific. Aqueous solutions of HCl and NaOH (purchased from Sigma-Aldrich) and deionized (DI) water (18.2 MΩ cm, Elga DI water system) were used in the experiments.

### 2.2 Preparation of 2T-Stabilized Pickering Emulsions

Membrane emulsification using a commercial system (LDC 1, Micropore Technologies, Redcar, UK) was used to prepare 2T-stabilized Pickering emulsion. This process has been reported in our previous work.^45^ In brief, 3 mL of sunflower oil was injected by a syringe pump (1002X, ProSense BV) into a chamber containing 27 mL of pH 7.0 buffer containing 0.5 mg/mL 2T. The emulsion was stirred by a paddle stirrer at a speed of 900 rpm, and the injection rate was 40 μL/min.

### 2.3 Characterization Methods

#### 2.3.1 Dynamic Light Scattering

Dynamic light scattering (DLS) measurements of the 2T dispersions in pH 7.0 buffer and pH 4.0 buffer were performed using a Malvern Zetasizer at an optimal temperature (25 °C). The instrument was fitted with a 4 mW 632.8 nm He–Ne red laser and a detector (avalanche photodiode) measuring the intensity of the scattered light positioned at 173°. Three individual measurements have been made 10 s after the dispersions were prepared. Each measurement is the sum of 12 runs, and each run takes 10 s.

#### 2.3.2 Morphological Observations

An optical microscope (Olympus BX53M), equipped with 10×, 20×, and 50× objective lenses were used to observe the microstructures of the Pickering emulsions. Samples were prepared by dropping several drops of emulsion on glass slides. Both reflected and transmitted light sources were used for observations. Digital images were analyzed using the analyze particle function with ImageJ software to find the diameter of all oil droplets. In the analysis routine, the scale and contrast were set, the background was subtracted, the image was binarized, and the cross-sectional areas were calculated.^46^ Origin 2020 software was then used to calculate the distribution of droplet sizes and the coefficient of variation (CV) defined as the standard deviation divided by the mean. At least two images were analyzed to determine the size distribution for each emulsion. For each image, at least 50 droplets were counted, and mean values are reported hereafter to represent the droplet size.

AFM (Dimension Edge, Bruker) was used to characterize 2T assemblies formed in pH 4.0 buffer and pH 7.0 buffer. To prepare samples, 2T dispersions in pH 4.0 or pH 7.0 buffer solutions (0.5 mg/mL) were spin-coated (PWM32 photoresist spinner, Headway Research) onto a silicon wafer at 4000 rpm. The silicon wafers were cleaned by ultrasonication for 15 min in acetone and water separately, followed by a UV–ozone treatment (Bioforce Nanosciences Inc., model UV.TC.EU.003) for 20 min before use. The quadratic roughness, *R*_q_, and the roughness factor were obtained from the AFM analysis of 10 μm × 10 μm images using Bruker software. Scanning electron microscopy (SEM, JSM-7100F, JEOL) was used to characterize the surface morphology of those samples for comparison. The typical accelerating voltage was 2 kV. Additionally, the morphologies of 2T films obtained from the oil/water interface were characterized by AFM and SEM.

#### 2.3.3 Small Angle X-ray Scattering

SAXS experiments on 2T solutions in DI water, pH 4.0 buffer, and pH 7.0 buffer were performed at the multipurpose X-ray instrument for nanostructural characterization (MINA) at the University of Groningen. The instrument is built on a high-intensity Cu rotating anode X-ray source, providing a parallel collimated X-ray beam with photon wavelength of λ = 0.1543 nm. The scattering patterns were collected using a 2D Vantec detector from Bruker placed 1 m away from the sample. The dispersions were contained in a sealed glass capillary of 1.5 mm outer diameter with 0.01 mm wall thickness. After subtraction of the scattering signal from the solvent background and radial integration from 2D patterns to 2D intensity profiles, the final intensity, *I*(*Q*) versus *Q*, curves were generated. The calibration of the sample-to-detector distance, the beam center position, and the probed angular scale was performed using the diffracted rings from a standard silver behenate powder sample.

For both measurements, the magnitude of the scattering vector (*Q*) is given by
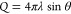
1where 2θ is the angle between the incident and scattered X-rays and λ is the wavelength of the incident X-rays. The characteristic lamellar periodicity (interlamellar spacing) is inversely proportional to the *Q* (100) peak position
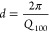
2

#### 2.3.4 Water Contact Angle Measurements

To characterize the wettability of 2T films obtained from the oil/water interface, the water contact angle was measured using a contact angle analyzer (Krüss, model DSA25B) at the room temperature of 21 ± 1 °C. 2T films were transferred from the oil/water onto a square piece of silicon wafer by two different methods: (1) pushing a wafer downward onto a floating film to produce a 2T surface presenting the water side and (2) lifting the wafer upward from under a floating film to produce a 2T surface presenting the oil side. On each sample, three 4 μL water droplets were deposited in different areas to acquire the mean water contact angle.

#### 2.3.5 Raman Spectroscopy

Raman spectra were obtained using the 473 nm excitation wavelength of an NTEGRA Raman microscope (NT-MDT, Moscow, Russia) equipped with a 60× objective lens. For detailed analysis, an 1800/600 grating was applied, resulting in an ∼1 cm^–1^ step size. The exposure time was 60 s for all measurements. Python code was used to correct the baseline.

#### 2.3.6 Grazing-Incidence Small Angle X-ray Scattering

Grazing-incidence SAXS (GI-SAXS) measurements on 2T films obtained from the oil/water interface were made on a Xenocs Xeuss 2.0 equipped with a Cu Kα source collimated by two sets of scatterless slits. A Pilatus 300k detector mounted on a translation stage was used to record the scattered signal. The distance between the detector and the sample was calibrated using silver behenate (AgC_22_H_43_O_2_), giving a value of 0.339(2) m. The GISAXS measurements were made with an incidence angle of 0.2°, which is just below the critical angle of 0.24° for glass. Two 20 min data collections were made with a small offset in the detector *z* position. These two images were then combined to produce a single image without any gaps between the detector chips.

### 2.4 Molecular Dynamics Simulations

MD simulations were carried out using the molecular operating environment software MOE 2019 suite^47^ to study (i) the self-assembly process of 2T in pH 7.0 buffer; (ii) the molecular arrangement of 2T lamellae in the liquid phase, and (iii) the mechanism of the stabilization of 2T at the oil/water interface.

To define an initial structure of 2T, C_10_(NGly_4_)_2_ was put together based on reported assembly features^35−37^ (“polyglycine II” nanostructures, in which peptide helices 3_1_ (ϕ = −76.9°, ψ = 145.3°) form a network of hydrogen bonds with six neighboring chains). The distance between two molecules was set to be 5 Å. Constraints on the dihedral (ϕ = −76.9°, ψ = 145.3°) have been applied.

The MD simulations were conducted by using the Nosé–Poincaré–Andersen (NPA) equations and Amber 14: EHT force field with a cut-off of 1.0 nm. Periodic boundary conditions were applied in all simulations. The system pressure was maintained at 1 atm, and the temperature was set to be 295 K. Before running a simulation, structure preparation and energy minimization functions were applied to ensure that the systems have an appropriate geometry. Energy minimization was set to be terminated when the root-mean squared gradient was lower than 0.1. Atomic coordinates were saved every 10 ps for the trajectory analysis. All restraints were removed before producing a simulation. In all simulation figures and videos, water molecules are hidden from view for a clearer visualization.

#### 2.4.1 Simulations of 2D Assembly and Molecular Conformation

Course-grained (CG) dynamic simulations using the MARTINI coarse-grained force field^48^ have been used previously to model the molecular self-assembly of supramolecular materials.^49^ In simulations here, the molecule C_10_(NGly_4_)_2_ was modeled by 11 beads (using MARTINI 3), as is shown in Figure S1a. The molecular volume and shape of the CG model was compared to that of the underlying all-atom (AA) structure (Gromacs force field) to verify the accuracy of the CG model. The solvent-accessible surface area (SASA) of the CG model and the corresponding AA model are shown in Figure S1b, and the values are calculated by using the Gromacs tool. The average AA SASA value is 10.43, while the average CG SASA value is 10.16. Thus, the average CG SASA value is only about 2.7% smaller than the AA value, which is in acceptable agreement.

128 molecules of the CG peptide were randomly inserted into a box of dimensions 10 × 10 × 6 nm^3^ and solvated with 2064 CG water molecules. Periodic boundary conditions were applied in the simulation. The system pressure was maintained at 1 atmosphere, and the temperature was set to be 300 K using the Berendsen algorithm. Before running a simulation, the box was equilibrated for 1,500,000 steps with a 25 fs time step. Then, 200 ns of CG dynamic simulation (Martini_v2.2 force field, timestep 10 fs) was performed. The first 40 ns of the simulation is shown in the Video S1 in the Supporting Information.

There are generally two possible alignments of C_10_(NGly_4_)_2_ to form a lamellar structure, which have been reported to be the U-shaped structure and a linear structure.^50^ However, there is no direct evidence of which one is truly how C_10_(NGly_4_)_2_ assembles in the liquid phase. To compare the stability of the two structures in liquid, an all-atom simulation was performed to clarify the conformation. 32 linear structures of C_10_(NGly_4_)_2_ and 32 U-shaped structures of C_10_(NGly_4_)_2_ were separately solvated in a water box using the “solvate” function in MOE. In the simulation box, 0.3 mol/L NaCl was added before the “solvate” step. The software generated 3986 water molecules with the set salt concentration. The size of the periodic cell was adjusted by the software based on the size of the 2T. The simulation was processed for 30 ns with a time step of 0.001 ps.

#### 2.4.2 Adsorption Process of 2T at the Oil and Water Interface

To build an oil and water two-phase system, 100 oil molecules were first added randomly into the box. After their energy minimization, the oil molecules were taken as an aggregate and were moved to the top of the box. To keep the oil phase at the top during the simulation, the top layer molecules of the oil were tethered. Then, the “solvate” function was used to add water molecules into the periodic cell. After the two-phase system was built, 2T was added and then a simulation was run. The simulated sunflower oil phase was composed of 30 triolein molecules, 65 trilinolein molecules, and 5 tripalmitin molecules,^51^ and the water layer was composed of 10234 water molecules and 0.3 mol/L NaCl. The cell size was adjusted manually to make sure that the density of the system was between 0.9 and 1 g/cm^3^. The simulation was processed for 10 ns with a time step of 0.0005 ps.

### 2.5 pH Responsiveness of 2T-Stabilized Pickering Emulsions

1 mL of solutions with different pH values (pH 2.0, pH 4.0, pH 6.0, and pH 9.0; adjusted with HCl or NaOH solutions) were dropped into transparent plastic chambers. Small volumes (25 μL) of 2T emulsions prepared in pH 7.0 buffer were dropped into the solutions and observed under the optical microscope. (The emulsion volume fraction in the mixture was low so that the final pH corresponded to the added HCl/NaOH solutions). A video camera connected to the optical microscope was used to record the emulsion response immediately after the buffer addition.

## 3 Results and Discussion

### 3.1 Self-Assembly of 2T in Aqueous Solution

**Figure 1:**
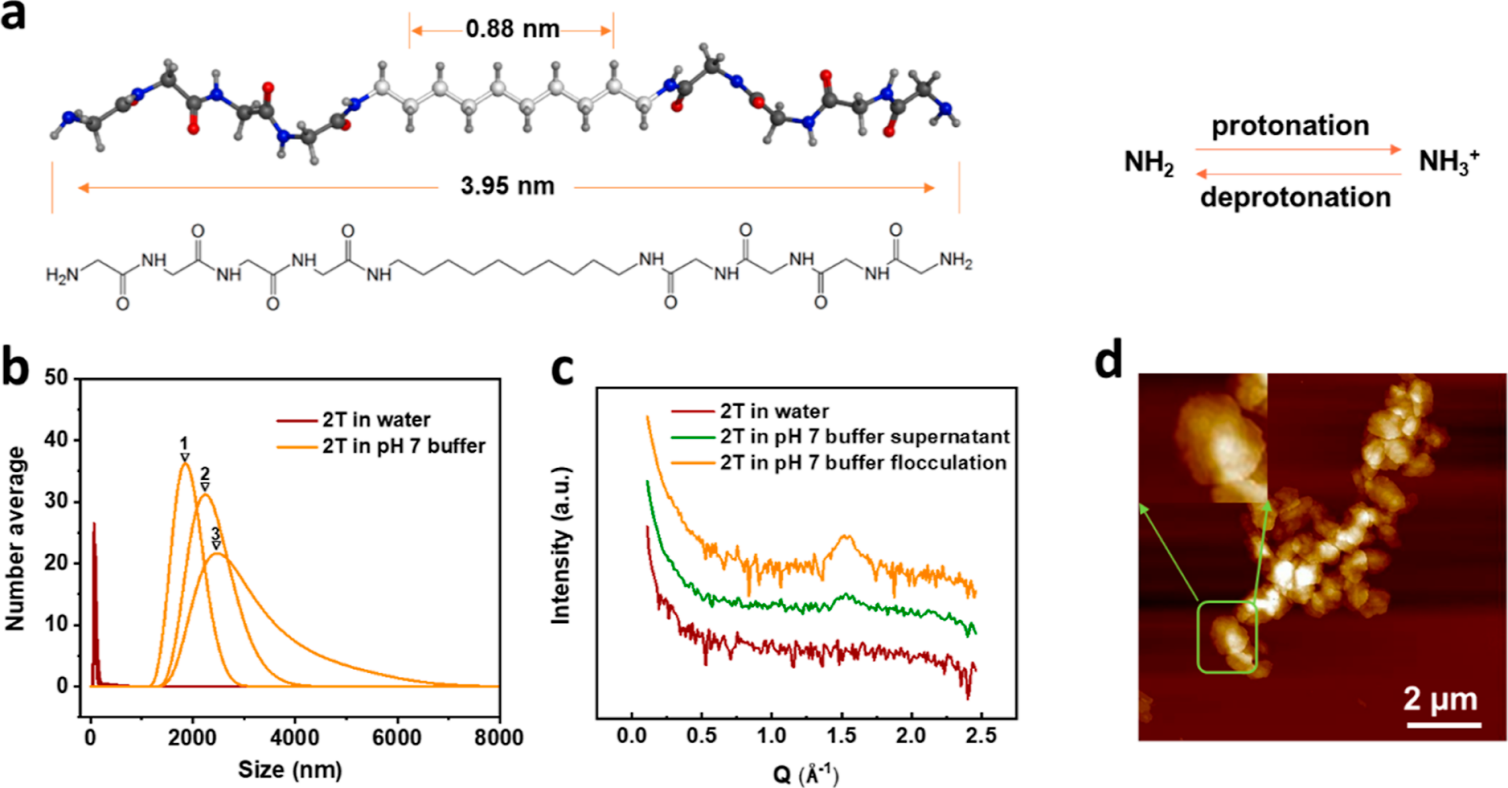
(a) Molecular model of C_10_(NGly_4_)_2_. (b) Number average size distributions (from DLS) for 2T in water and pH 7.0 buffer, taken at three successive times. (c) SAXS data for 2T in water and pH 7.0 buffer. The data are shifted vertically for clarity. (d) AFM topographic image of 2T after spin-coating on silicon wafer from a pH 7.0 buffer solution.

The molecular structure of C_10_(NGly_4_)_2_ is presented in Figure 1a. The structure of C_10_(NGly_4_)_2_ (hydrophobic alkyl chain and hydrophilic peptide antennae) explains its amphiphilic nature.^38,41^ With protonation, the terminating amino groups can be changed from NH_2_ to NH_3_^+^ following a pH decrease and deprotonated back to NH_2_ with a pH increase.

DLS was carried out to investigate the spontaneous molecular assembly of 2T in aqueous solution. According to the number average size distribution obtained through DLS (Figure 1b), the size of 2T in DI water was always between 70 and 100 nm for three successive time measurements. In contrast, when the 2T was dissolved into a pH 7.0 buffer, the peak size was larger and increased over time (1862, 2235, and 2480 nm on successive measurements). Over the time of the measurement, there was continuous growth and sedimentation. The size distribution in the pH 7.0 buffer in the three successive measurements was far broader than when in DI water. The size distribution in pH 4.0 buffer was also studied by DLS for comparison (see Figure S2a).

It is known that C_10_(NGly_4_)_2_ can be well dispersed in DI water, and the pH value of the final dispersion is 5.8 when the concentration is 0.5 mg/mL.^42^ In pH 7.0 buffer, deprotonation of terminal amino groups and also screening of the charge due to the presence of phosphate ions in the buffer reduce the repulsive forces and lead to the formation and sedimentation of large aggregates. In pH 4.0 buffer, the average size of 2T is smaller than in the pH 7.0 buffer, as the protonation of terminal amino groups increases electrostatic repulsive forces. This finding is consistent with visual observations of 2T in pH 4.0 and pH 7.0 buffer solutions (Figure S2a,e–h). 2T dispersions appear cloudy in pH 7.0 buffer with some visible structure, but they remain clear in pH 4.0 buffer.

To obtain information on the possible molecular ordering of 2T in the emulsions, SAXS was performed for the first time on 2T structures both in DI water and in pH 7.0 buffer. (In the buffer, both the supernatant and sediment were measured.) In the SAXS data obtained from 2T in DI water, there is no evidence of structure formation (Figure 1c). However, for 2T in pH 7.0 buffer, there is a clear scattering peak centered at 0.140 Å^–1^ for the 2T sediment. The scattering peak suggests the existence of a structure with stacking periodicity of 4.49 nm being formed in the pH 7.0 dispersion. With time, the system tends to flocculate and deposit toward the bottom of the capillary, concentrating the scattering objects, and making the scattering peak more visible yet unaltered (Figure 1c). Comparing the results at pH 7.0 in buffer with the results in DI water reveals that there is an effect of the ionic concentration. The presence of ions in the buffer solution will screen repulsive charges to allow assembly, whereas in water, charge repulsions will oppose assembly.

When 2T in pH 7.0 buffer was spin-coated on a silicon wafer, AFM imaging (Figure 1d) found overlapping platelets whose sizes ranged from 300 nm to 1 μm (according to image analysis). Some individual platelets had a thickness of ca. 15 nm and presented a terraced surface with a step height of approximately 5 nm. Also, platelets adsorbed from solution onto mica surface were observed (Figure S2b). The solution’s SAXS results together with the AFM observations suggest a stacked, lamellar-like assembly of 2T, as will further be confirmed by GISAXS analysis of the solid, dry state below.

In addition, SAXS on 2T in a pH 4.0 buffer shows an amorphous structure (Figure S2c), and AFM shows irregular distorted structures for 2T spin-cast from pH 4.0 solutions (Figure S2d). Comparison of the structure at pH 4.0 to that at pH 7.0 in buffer shows the effect of pH (rather than ionic concentration) in triggering disassembly.

Although SAXS analysis found evidence for a lamellar structure, the specific molecular ordering cannot be determined from SAXS alone. The use of structural parameters directly accessible by SAXS, together with input from MD simulations, is a powerful tool to achieve detailed knowledge of molecular arrangement in self-assembling organic materials.^52,53^

**Figure 2:**
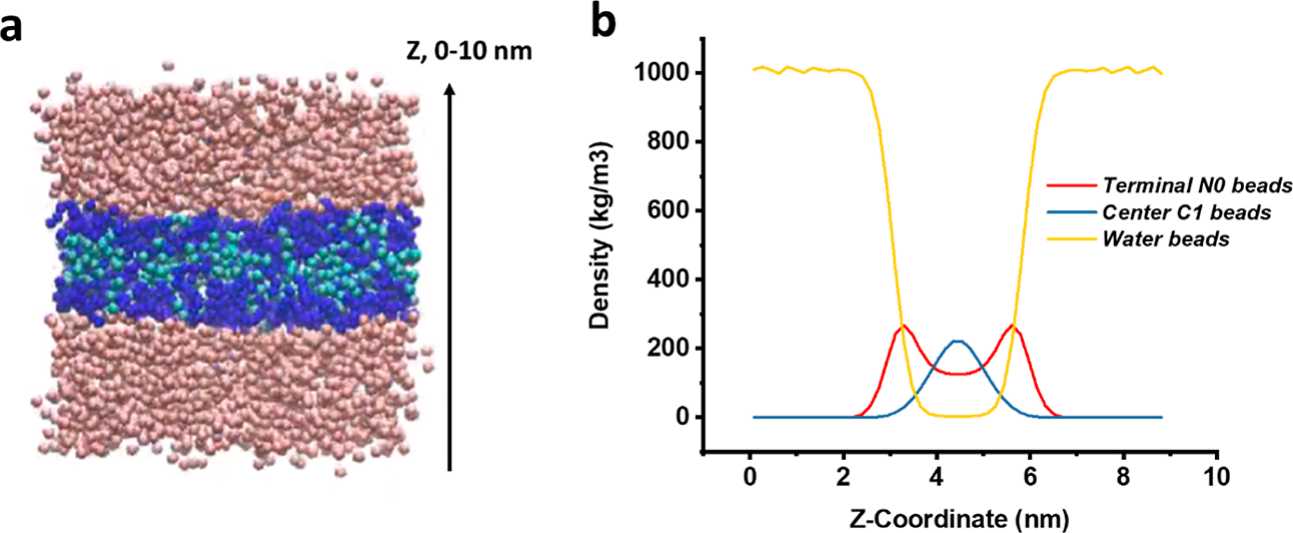
(a) Snapshot of a frame at 40 ns in a coarse-grained dynamic simulation. The three CG beads are water (pink), terminal N0 (blue), and C1 (cyan). (b) Density profile of the three beads in the snapshot, showing the terminal N0 aligned at an interface with water, and the C1 beads sandwiched in the center of the self-assembled structure.

The formation process of the plate structures was investigated via coarse-grained simulations (Figure 2). From the simulation video (see Supporting Information Video S1), we can see the formation process of the assembly structures. All CG peptides accumulated and aligned at the box center. A frame obtained after 40 ns of the simulation (Figure 2a) was chosen for density analysis. The density profiles of three different CG beads (water, the terminal glycine residue (N0), and the central C_4_H_8_ segment (C1) as defined in Figure S1a) are presented in Figure 2b. We see that a lamellar structure has spontaneously assembled by this time point. No water appears inside the assembled structure. The density of the C1 beads is highest in the central layer, while the terminal N0 beads align at the two interfaces with the water.

**Figure 3:**
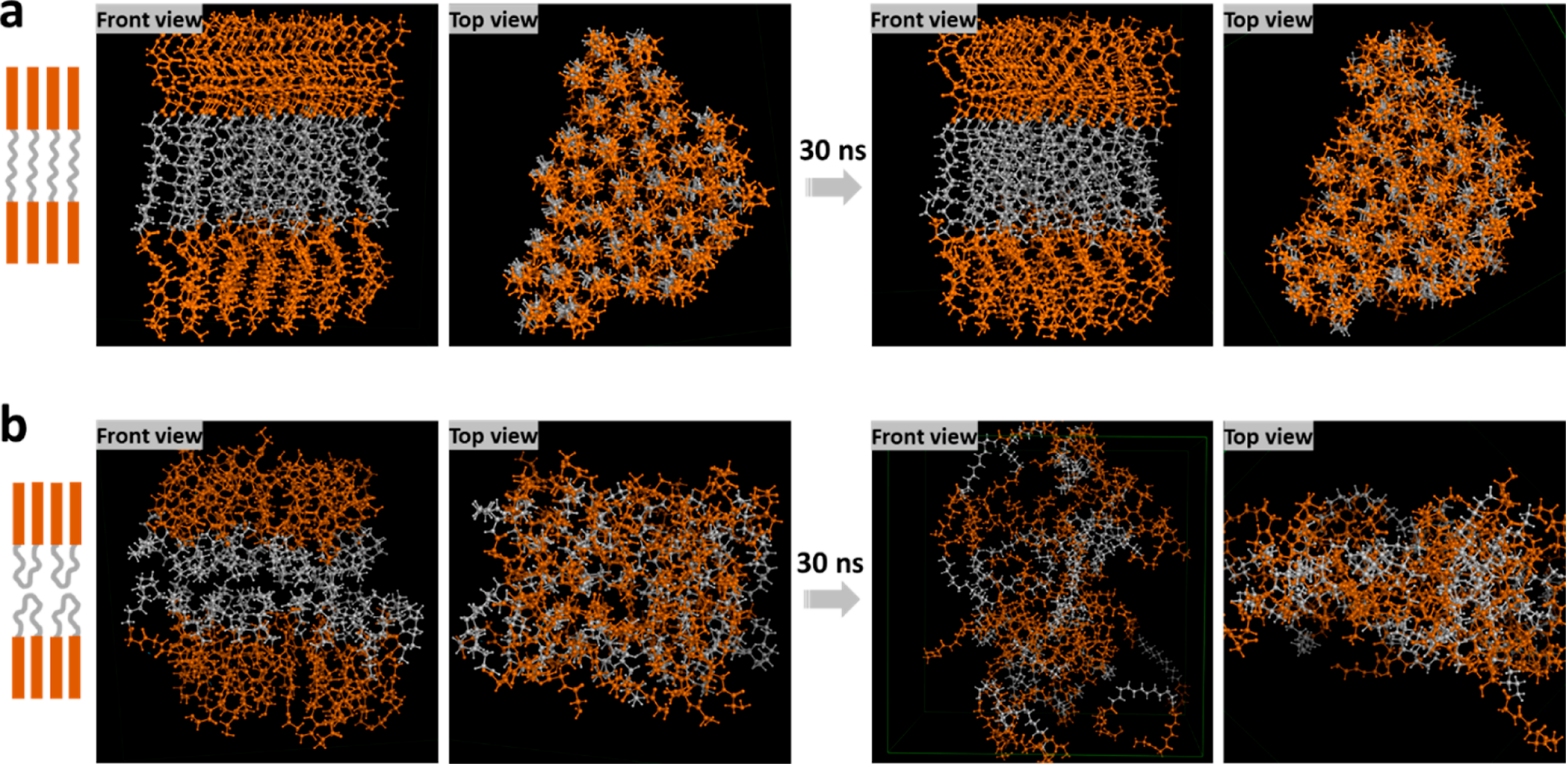
(a) Front and top views of linear aligned 2T before and after simulations. (b) Front and top views of U-shaped 2T before and after simulation.

The advantage of coarse-grained simulation is that it can greatly reduce the calculations and speed up the simulation process. However, it is difficult to investigate the alignment of the C_10_(NGly_4_)_2_ molecule and the conformation of the assembly structure. Therefore, an all-atom simulation was performed. The two most probable structures (linear and U-shape) are shown in the diagrams on the left side of Figure 3a,b, respectively. To understand which one is favored in the liquid phase, MD simulations were performed to compare the stability of the two structures. During the 30 ns simulation, the linear aligned 2T is stable. Essentially, no significant changes can be found in the assembly, as seen from both the top view and the front view. However, in the U-shaped layered 2T structure, the molecules were very mobile during the simulation time. Finally, they formed an irregular 2T aggregate at the end of the simulation.

The dimensions of the simulated structures can also be used to interpret the SAXS data. As is shown in Figure S3, the model’s length of the linear molecule is 3.95 nm, while the value for U-shape conformation is only 3.49 nm. According to these simulations, the linear structure is closer to the lamellar distance of 4.49 nm found from SAXS for the 2T in pH 7 buffer solution. The small difference in dimensions could be due to the dislocation of C_10_(NGly_4_)_2_ in the assembly or the existence of a water layer between the lamellae. Therefore, we conclude that it is most likely that C_10_(NGly_4_)_2_ is linearly aligned in the liquid phase.

### 3.2 Analysis of 2T at the Oil/Water Interface

**Figure 4:**
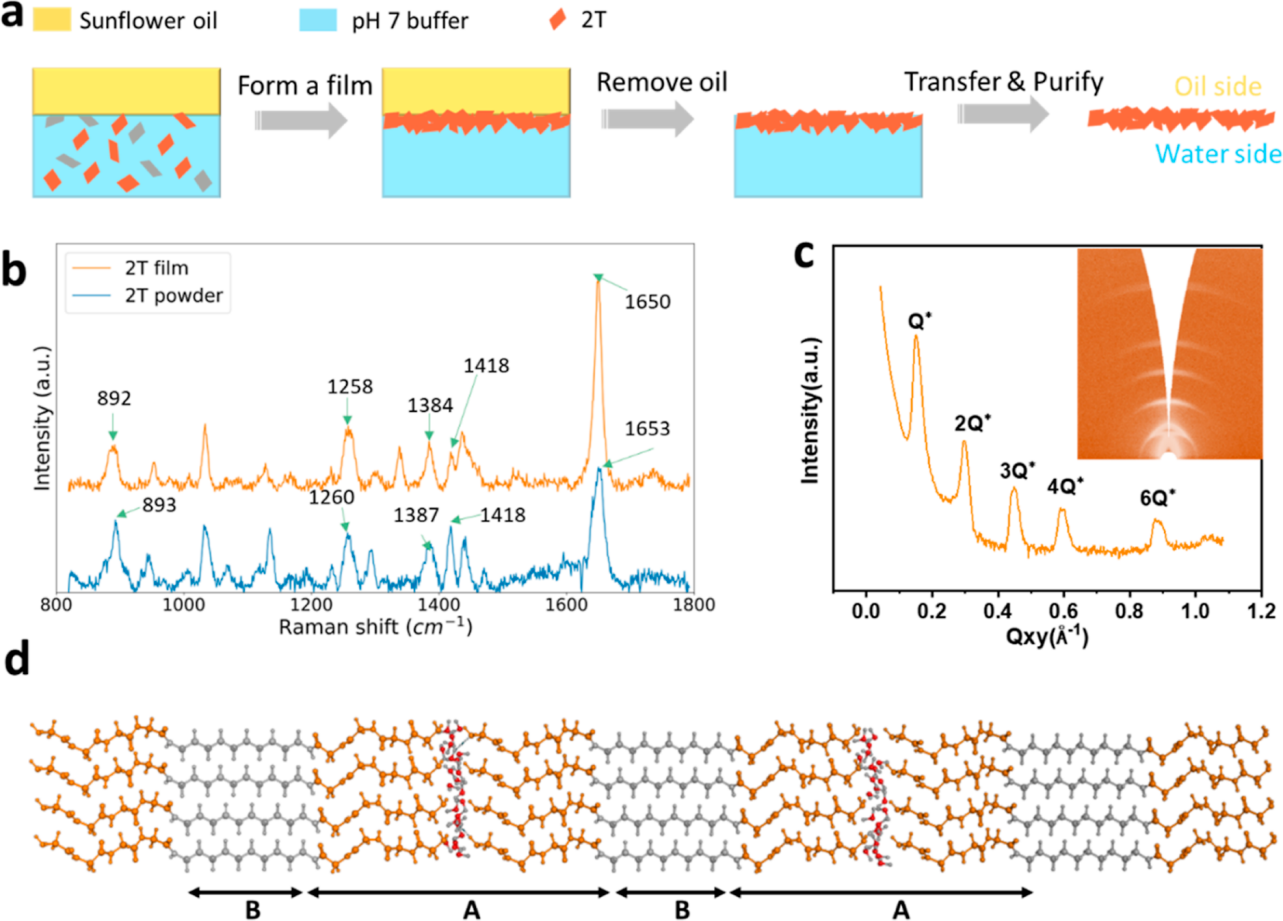
(a) Schematic diagram showing how a 2T film was deposited from the oil/water interface. 2T in pH 7.0 buffer adsorbs at the oil/water interface, after which it is transferred to a substrate and purified. (b) Raman spectroscopy of the as-received C_10_(NGly_4_)_2_ powders and a dry 2T film. (c) Out-of-plane GISAXS data obtained from a dry 2T film. The inset shows the *Q*_z_ versus *Q*_xy_ map. The masks used during integration are shown in white. Analysis found an ABAB structure with *A* = 3.36 nm and *B* = 0.83 nm. (d) Molecular model showing an edge view of an ABAB lamellar structure with *A* = 3.11 nm and *B* = 0.88 nm. A water layer is included between the lamellae, such that the distances are consistent with GISAXS.

To study the properties of 2T adsorbed at the oil/water interface, a 2T film was collected onto a silicon wafer, following the procedure shown in Figure 4a. The deposited film was wetted with nonvolatile oil (Figure S4a), which made it difficult for further characterization. Toluene, in which the oligoglycine is not soluble, was used to purify the 2T film, which was then transferred to a silicon substrate. An optical photograph of the purified 2T film is shown in Figure S4b. The color of the film was white after the oil and water were removed.

Raman spectra of a purified 2T film and the as-received C_10_(NGly_4_)_2_ powder are shown in Figure 4b. According to Small et al.’s report,^54^ for a polyglycine type II structure, Raman peaks at 1654 cm^–1^ for amide 1 band, 1421 cm^–1^ for CH_2_ bending, 1383 cm^–1^ for CH_2_ wagging, 1261 cm^–1^ for CH_2_ twisting, and 884 cm^–1^ for CH_2_ rocking could be found. The polyglycine type II structure of 2T has already been confirmed by Tsygankova and co-workers,^50^ where they identified those peaks with very small differences in 1424 cm^–1^ for CH_2_ bending and 1382 cm^–1^ for CH_2_ wagging. In this work, as is shown in Figure 4b, the specified peaks obtained from 2T films are also close to the peaks from the literature with minor shifts (1653, 1418, 1387, 1260, 893 cm^–1^ for C_10_(NGly_4_)_2_ powders and 1650, 1418, 1384, 1258, 892 cm^–1^ for the 2T film). The minor differences may arise from the different methods of preparing samples. Based on the presence of those characterization peaks, it can be concluded that the 2T structure organization in films corresponds to the polyglycine type II structure, and the purification process with toluene to make a film does not change the structure. Raman spectroscopy of pure sunflower oil was also performed (Figure S5) to determine the characteristic peaks. The disappearance of a strong peak at 1087 cm^–1^ following the rinsing of the 2T films with toluene indicates the removal of the oil phase.

The structure of the purified 2T film was determined by GISAXS. As is shown in Figure 4c, the *Q* position of the first peak is at a value of 0.150 Å^–1^. This scattering pattern is consistent with a lamellar structure. The higher-order peaks appear at the expected positions of Q*, 2Q*, 3Q*, 4Q*, and 6Q* for a lamellar structure. GISAXS data analysis found that the lamella was made of an ABAB type of structure, with *A* = 3.36 nm and *B* = 0.83 nm, for a total lamellar distance of *d* = 4.19 nm (see Supporting Information). Compared to the lamellar thickness of 4.49 nm for the 2T assembly in pH 7.0 buffer obtained from SAXS (Figure 1c), there is a very small difference of 0.3 nm, which may be due to the influence of a water layer between the lamellae for 2T in solution (Figure 4d).^55^

As is shown in Figure 4d, the thickness of the A-layer in the lamellar structure, as obtained from GISAXS (3.36 nm) is comparable to the length of two antennae for the oligoglycine stacked end-to-end (3.07 nm), whereas the B-layer (0.83 nm) is comparable to the length of the central alkyl chain (0.88 nm), according to the modeling (Figure 1a). The lamellar thickness values obtained both from GISAXS and SAXS experiments are slightly greater than that found in the molecular modeling, which can be explained by a broadening of the layers because of imperfect registry.

**Figure 5:**
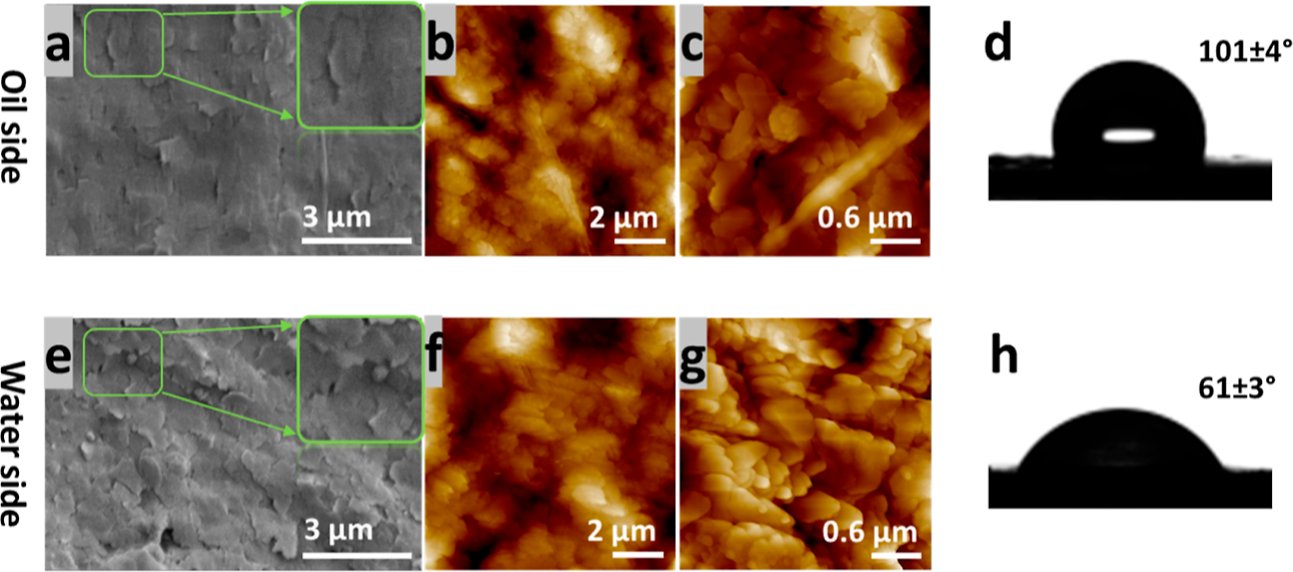
(a) SEM and (b,c) AFM topographic images showing the morphology of the 2T film from the oil side. (d) Sessile water drop and contact angle of the 2T film on the oil side. (e) SEM and (f,g) AFM topographic images showing the morphology of a 2T film from the water side. (h) Sessile water drop and contact angle of the 2T film on the water side.

The morphology of the 2T films from both the oil side and water side were studied by SEM and AFM. By comparing Figure 5a–c with Figure 5e–g, we can see that the “2T platelets” on the oil side tend to be wider and flatter, while for the water side, the “2T platelets” are more separated. To quantify these differences, the roughness and roughness factor for the oil-side film was found to be 108 nm and 1.08, respectively, whereas for the water-side film, the values are 162 nm and 1.15, respectively. The water contact angles of both sides of the films are presented in Figure 5d,h. The water contact angle value on the oil side is higher than on the water side. As the roughness factors for both sides are relatively low, the surface topography cannot explain the large differences in the water contact angle values.^56^ Hence, we presume that the higher water contact angle (lower surface energy) arises from the partial re-organization of the C_10_(NGly_4_)_2_ molecules on the oil side of the interface. The hydrophobic alkyl chain of 2T will be exposed to the oil phase, rendering hydrophobicity to that side of the film.

**Figure 6:**
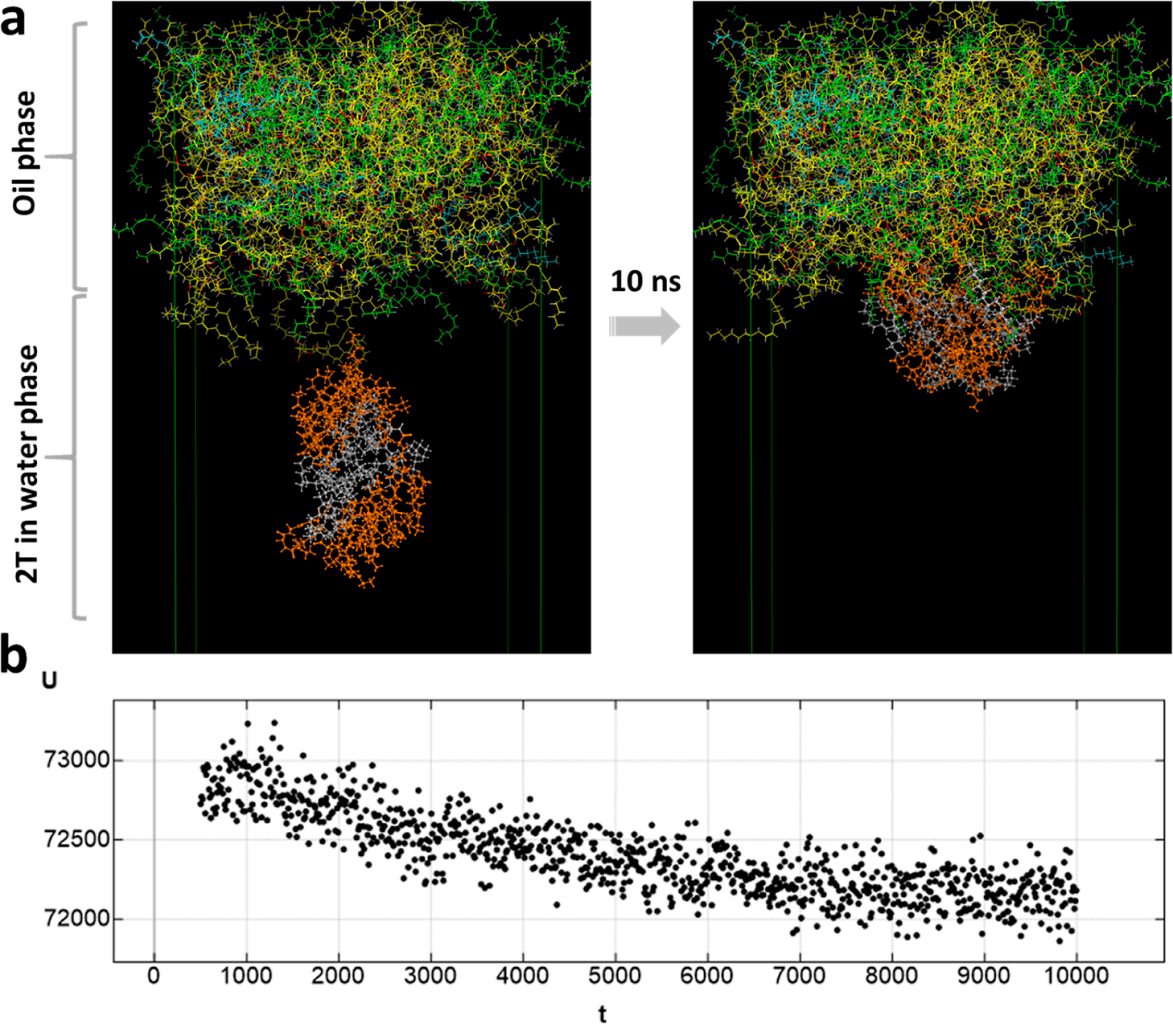
(a) Snapshots of structures in MD simulations of 2T attaching to the oil/water interface. (b) Potential energy during a 10 ns simulation of 2T at the oil/water interface (the time, *t*, on the *x*-axis is given in units of ps).

To probe the spontaneous adsorption of 2T at the oil/water interface, we performed a MD simulation, shown in Figure 6 and in a video (see Video S2 in Supporting Information). The 2T assemblies gradually moved toward the oil and water interface. Over the 10 ns of simulation, the potential energy of the system gradually decreased and then reached equilibrium, which is consistent with the minimum energy principle. Hence, the simulations suggest that the 2T assemblies will be favorable as a Pickering 2D stabilizer.

### 3.3 Use of 2T as Pickering Stabilizers and Emulsion pH Responsiveness

**Figure 7:**
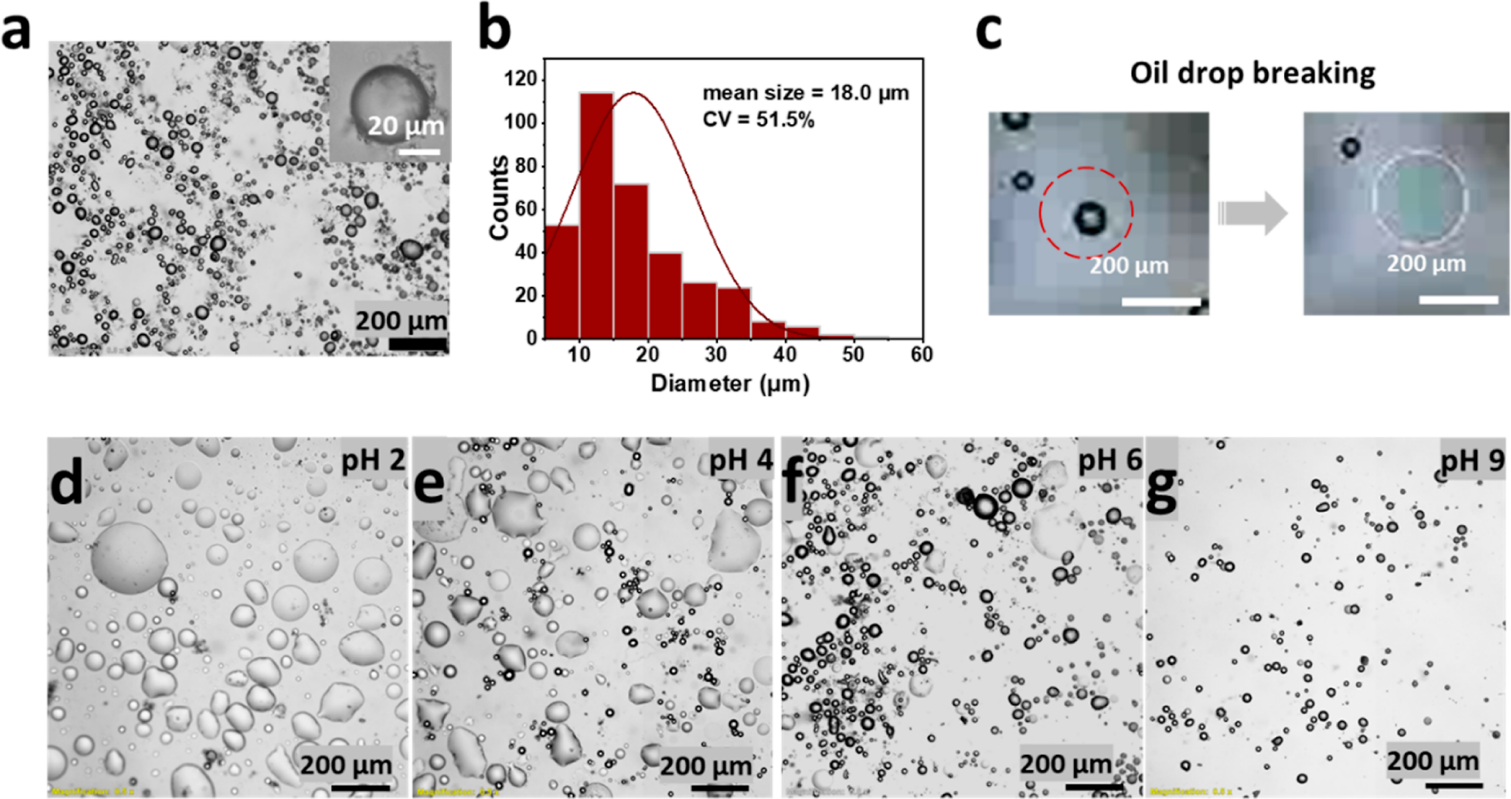
(a) 2T-stabilized Pickering emulsion prepared by membrane emulsification in pH 7.0 buffer. The inset shows a single oil droplet. (b) Size distribution of the oil drops in the emulsion obtained from image analysis. (c) Optical microscope images showing the pH response when a single 2T-stabilized oil drop was added to a pH 4.0 buffer solution. The red dashed circle shows the oil drop before breakage. Optical microscope images show 2T emulsions that were added to solutions with different pH: (d) pH 2.0; (e) pH 4.0; (f) pH 6.0; and (g) pH 9.0 (adjusted using HCl or NaOH).

After determining the fundamental properties of 2T in the liquid phase, as well as 2T at the oil/water interface, we investigated the use in emulsions. For the first time, we produced 2T-stabilized Pickering emulsions by using membrane emulsification. A stable emulsion in pH 7.0 buffer with a mean size of 18 μm is shown in Figure 7a,b. There was some very slow coalescence during storage. It took 6 months for the mean size to be doubled to 37.2 μm (Figure S7c,d).

Note that a stable emulsion could not be produced when using DI water (with or without 2T) as the continuous phase. Measurements of the interfacial tension found evidence for only weak interfacial activity of 2T (i.e., a decrease in the interfacial tension of 2.6 mN/m), suggesting that there is not enough adsorbed to stabilize the emulsion as a conventional surfactant (see Figure S7a). Moreover, a stable emulsion could not be obtained in the pH 7.0 buffer without added 2T.

We have already shown here that 2T shows different structures when the pH of the solution is changed. For the corresponding 2T emulsions, we have found a pH response in the emulsion stability. Thus, when 2T-stabilized oil drops are added into a pH 4.0 buffer solution, they immediately become unstable. The cross-sectional area of the oil drop grows larger, and the oil spreads on the top of the water phase (Figure 7c). To investigate the pH response further, 2T emulsions were added to a series of pH solutions (pH values of 2.0, 4.0, 6.0, and 9.0) adjusted using HCl or NaOH, as shown in Figure 7d–g. Almost no broken oil drops can be found in the pH 9.0 solution; the emulsion remains stable. However, if 2T oil emulsions are placed into an acid solution, the oil drops break. With greater acidity, the emulsion is destabilized faster and to a greater extent. This result can be explained by the protonation of the amino groups in C_10_(NGly_4_)_2_ at a lower pH, which creates a repulsive force between those molecules, ultimately destabilizing the lamellar structure and the emulsions. When the solution was adjusted back to pH 7.0, the oil droplets were not recovered.

The destabilization of the emulsions triggered by a pH change is a useful function with applications in drug delivery and controlled release of active ingredients. We illustrate here how the pH-responsiveness of a 2T-stabilized Pickering emulsion can be used in controlled release. β-Carotene (as a model active ingredient) was added to the oil phase before its emulsification with 2T (see Figure S8). When the pH was lowered via HCl addition, the emulsion was destabilized. A cream layer was converted to an oil layer within a time period of 1 min, and the β-carotene was no longer confined within droplets in the water phase.

**Figure 8:**
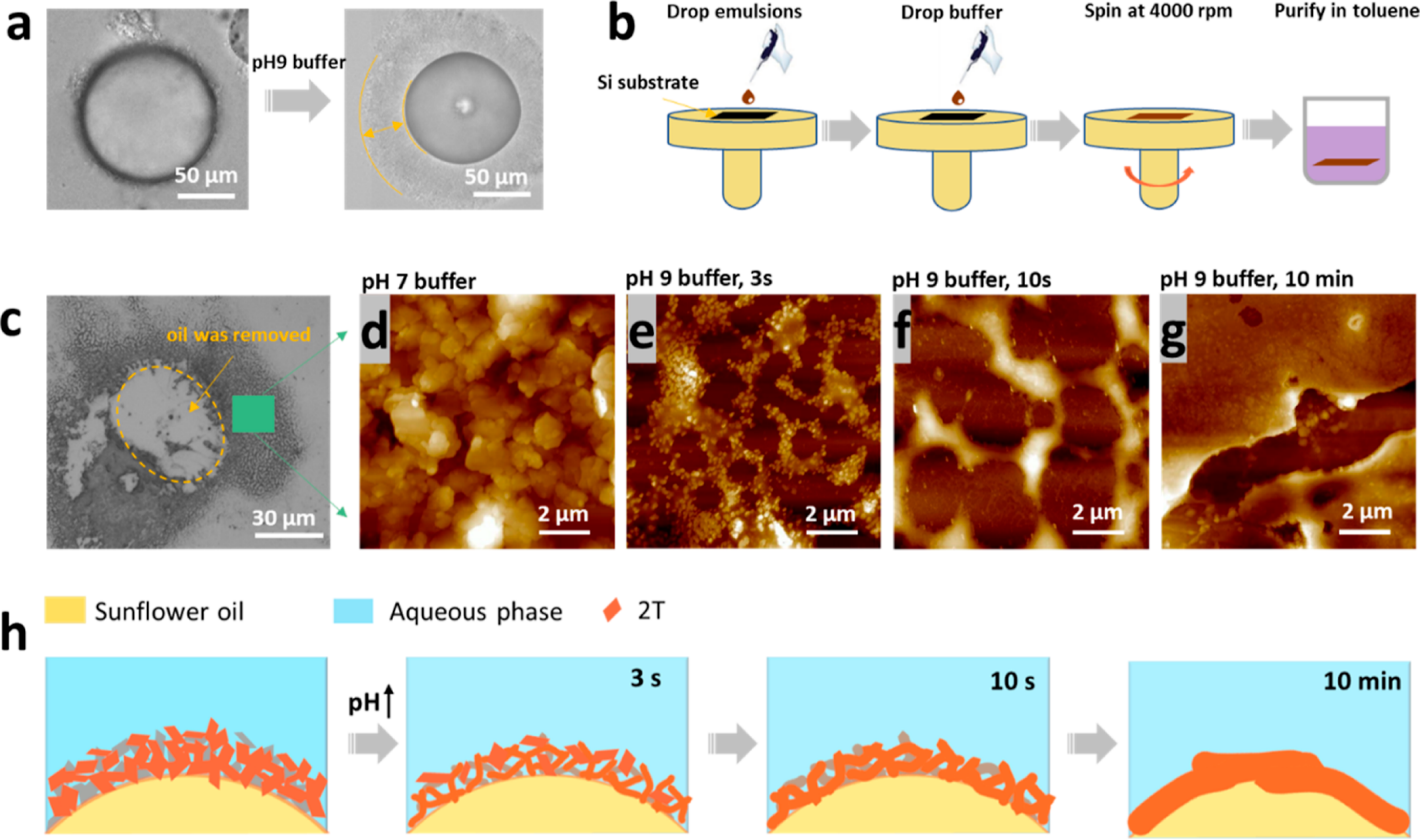
(a) Optical microscope images showing that, in pH 9.2 buffer, the morphology of the emulsion oil droplets evolved over time and an “egg cell” structure is formed. (b) Drawings of the process of spin-casting 2T emulsions onto a silicon substrate and purification from 2T emulsions treated with a pH 9.2 buffer. (c) Optical microscope image of the 2T structure after spin-coating and purification. (d–g) AFM images of 2T structures obtained from 2T emulsions treated with a pH 9.2 buffer over different times: 3 s, 10 s, and 10 min. (h) Schematic diagram showing the evolutionary process of the 2T emulsion wall structure over time.

Interestingly, we found that the morphology of the emulsion oil droplets upon treatment in pH 9.2 buffer evolved over time (with storage times of 3 months or more). Thus, Figure 8a shows that when the 2T emulsion was dropped into a pH 9.2 buffer solution with a 1:1 volume ratio, a thick capsule layer of around 20–50 μm is formed, giving the structure of the oil droplet the appearance of a human egg cell. (The resulting pH was measured to be 8.7 when mixing the pH 7.0 and pH 9.2 buffers by equal volume.)

In our experiments, we found that adding more pH 9.2 buffer (higher than a 1:1 ratio) still produced this “egg cell” structure. However, when the pH was adjusted to higher than 11.0, the encapsulating 2T layer (i.e., the “cell wall”) was separated from the oil drop surface. A minimum storage time was needed to allow the structure to form. The time of 3 months or longer was found to produce better defined egg cell structures. If the storage time was less than 1 month, the egg wall was not very clear to observe. There was insufficient time for the 2T to accumulate at the interface.

We presume that the “egg wall” layer surrounding the oil phase arises from the reorganization and accumulation of the C_10_(NGly_4_)_2_ molecules. To better study this interesting structure, we followed the procedure presented in Figure 8b, that is, spin-coating on a silicon substrate and purification by toluene rinsing, to cast the “egg wall” capsule on a silicon substrate, as is shown in the microscope image in Figure 8c and SEM image in Figure S9. The evolution of the oil droplets with time can be seen in the AFM images in Figure 8e–g. Thus, when 2T emulsions (in pH 7.0 buffer) were cast on the substrate, platelets of the lamellar structures, which had been lying at the original oil/water interface, were apparent (Figure 8d). When the emulsion was mixed with the pH 9.2 buffer for only 3 s before spin-coating, the lamellae were partially fused and textured structures were formed (Figure 8e). When the exposure time of the pH 9.2 buffer was increased to 10 s before spin-coating, larger textures were observed (Figure 8f). When the exposure time was as long as 10 min, thickened shells were observed (Figure 8g), indicating that the 2T lamellae were fused. This evolutionary process of the 2T platelets at the oil/water interface in the emulsion is probably resulting from the long-time accumulation of C_10_(NGly_4_)_2_ at the interface and a pH-enabled fusion. A reduced charge in C_10_(NGly_4_)_2_ resulting from further deprotonation of the terminating amino group in a pH 9.2 buffer would lead to reduced repulsion between the lamellar platelets. Therefore, larger textures are formed gradually. This process is represented by the diagram in Figure 8h.

The “egg cell” emulsion was dropped on silicon and dried within 5 min. Thereafter, energy-dispersive X-ray spectroscopy (EDS) was used to analyze the elemental content, going from the center of the oil drop to the edge of the “egg wall” corona, as is shown in Figure S10. We found that there was more elemental P and Na from the buffer in the corona nearer to the edge of the oil drop. This result indicates that the “egg wall” allows ions to transfer from the outer aqueous phase to the oil interface.

## 4 Conclusions

A novel lamellar structure of the triblock peptide amphiphile, C_10_(NGly_4_)_2_, has been found in pH 7.0 buffer solutions and also at the interface of O/W emulsions. Through MD simulations, it was shown that the linear alignment of C_10_(NGly_4_)_2_ molecules is stable in the liquid phase, whereas the U-shape conformation is not. The length of a linear C_10_(NGly_4_)_2_ molecule (3.95 nm), according to modeling, is close to the repeating distance of the lamella (4.19 nm) obtained via the first-reported use of GISAXS on a purified and dried film collected from 2T adsorbed at the oil/water interface. In contrast, the height of a U-shaped C_10_(NGly_4_)_2_ molecule from modeling is only 3.49 nm, which does not agree with the lamellar distance. SAXS showed that the lamellar thickness of 2T in pH 7.0 buffer solution was greater (approximately 4.49 nm) than in the dried film, which is presumed to be because of water being included in between the layers.

2T lamellar structures were demonstrated for the first time as a Pickering emulsion stabilizer. Stable 2T emulsions were produced in pH 7.0 buffer by membrane emulsification. These emulsions showed only very slow coalescence during storage for 6 months. Moreover, the emulsions showed a strong pH response in an acidic environment. Even when the pH of the water phase was only slightly reduced from pH 7.0 to pH 6.0, the oil droplets became less stable. This effect is explained by the protonation of the terminating amino group of the 2T, which imparts inter-molecule repulsions, which destabilizes the lamellae and hence the emulsions. Emulsions could not be formed using 2T in DI-water when the amphiphilic peptide exists as isolated molecules.

Interestingly, we found that, when treated with pH 9.2 buffer, the morphology of the emulsion oil droplets evolved over time and an “egg cell” structure was observed. AFM images disclosed the evolutionary process to form larger textures as 2T lamellae gradually fuse.

This paper delivers a thorough understanding of the formation of novel Pickering emulsions using 2D self-assembled peptide amphiphiles. The emulsions are a precursor to make more advanced structures. In contrast with other Pickering emulsifiers, such as GO or MoS_2_, triblock peptide amphiphiles enable the formation of biocompatible stable emulsions. The rapid disintegration of the emulsions in acidic environments (as demonstrated here in β-carotene emulsions) opens up a plethora of new and exciting potential applications in cosmetics, pharmaceuticals, and even tissue engineering fields. One of the applications of the 2T Pickering emulsions could be in ocular or nasal drug delivery, as a penetration enhancer,^57^ in instances where conventional surfactants are irritating or toxic. Hybridization of 2T nanosheets with other nanoparticles, such as nanodiamonds or GO (although beyond the scope of this work), could provide additional functionalities and tunable properties. Membrane emulsification is a low-energy, scalable process that offers an attractive means to fabricate emulsions at larger quantities. Ultimately, we developed a novel materials system to making Pickering emulsions which can be significant for biomedical and pharmaceutical applications.

## Supporting Information Available

Size, SAXS, and microscope images of 2T in pH 7.0 buffer, water, and pH 4.0 buffer; description of coarse-grained simulations; molecular model; photograph of the 2T films from the oil/water interface; Raman spectrum of pure sunflower oil; interfacial tension measurements; aging of the emulsion; destabilization of a β-carotene emulsion; SEM image of the spin-coated emulsion; and EDS measurements of the elements in a dried 2T-stabilized oil drop (PDF)
Coarse-grained simulation of the peptide in water, demonstrating the formation process of the 2D peptide assembly (MP4)
All-atom simulation of 2T attaching to the oil/water interface (MP4)
