# Supplementary material for: Two-Dimensional Triblock Peptide Assemblies for the Stabilization of Pickering Emulsions with pH Responsiveness
Source: ACS Appl Mater Interfaces. 2022 Nov 15;14(47):53228–40. doi: 10.1021/acsami.2c17558 (PMC9716523; doi:10.1021/acsami.2c17558)
Supplement: Supplementary file 1 — am2c17558_si_001.pdf [file am2c17558_si_001.pdf]

Two-Dimensional Triblock Peptide Assemblies for the Stabilization of Pickering Emulsions with pH Responsiveness

Zhiwei Huang,[a] Eleonora Calicchia,[b,c] Izabela Jurewicz,[a] Edgar Muñoz,[d] Rosa Garriga,[e] Giuseppe Portale,[c] Brendan J. Howlin,[f] and Joseph L. Keddie[a, *]

[a] Department of Physics, Faculty of Engineering and Physical Sciences, University of Surrey, Guildford, GU2 7XH, UK.

[b] Groningen Research Institute of Pharmacy, University of Groningen, A. Deusinglaan 1, 9713 AV Groningen, The Netherlands.

[c] Zernike Institute for Advanced Materials, Faculty of Mathematics and Natural Sciences, University of Groningen, Nijenborgh 4, 9747AG, Groningen, The Netherlands.

[d] Instituto de Carboquímica ICB-CSIC, Miguel Luesma Castán 4, 50018 Zaragoza, Spain.

[e] Departamento de Química Física, Universidad de Zaragoza, 50009 Zaragoza, Spain.

[f] Department of Chemistry, Faculty of Engineering and Physical Sciences, University of Surrey, Guildford, GU2 7XH, Surrey, UK.

E-mail: j.keddie@surrey.ac.uk (Joseph L. Keddie)

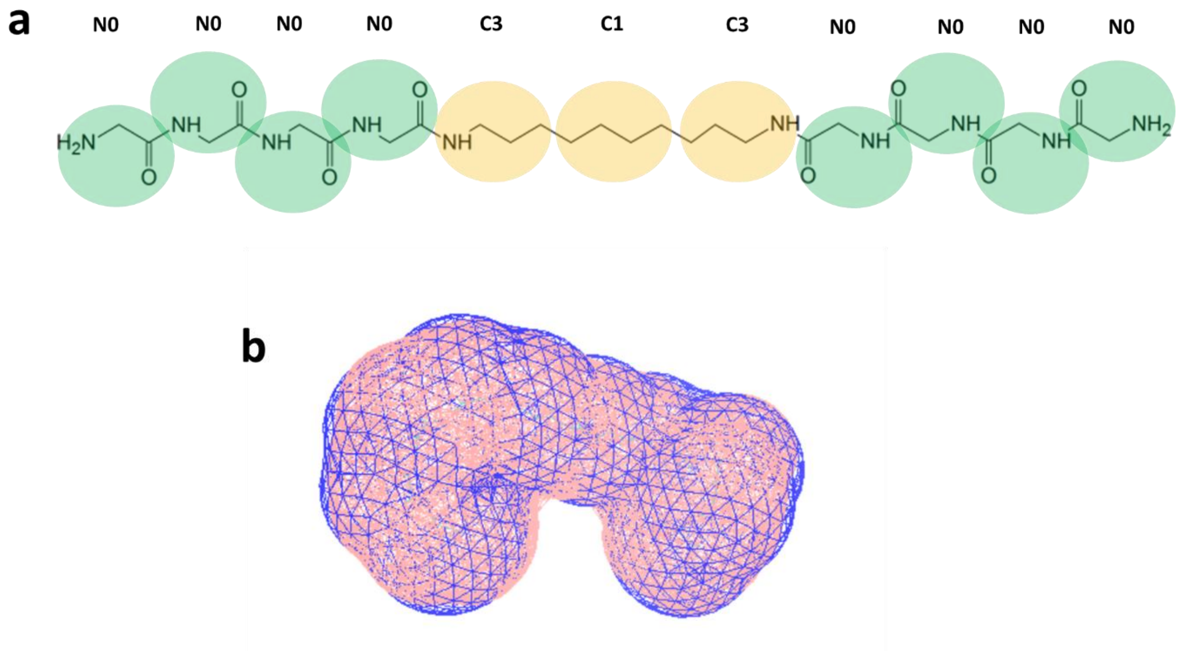

Figure S1. (a) Schematic structure of the all-atom and CG model of $C_{10}(NGly_4)_2$ peptide. Bead types are given above the CG model. The beads correspond to the glycine residues (designated as N0), a central C_4H_8 segment (C1), and C_3H_6NH (C3). (b) The solvent accessible surface area (SASA) of the CG model is shown using pink gridlines, and the AA model is shown using blue gridlines.

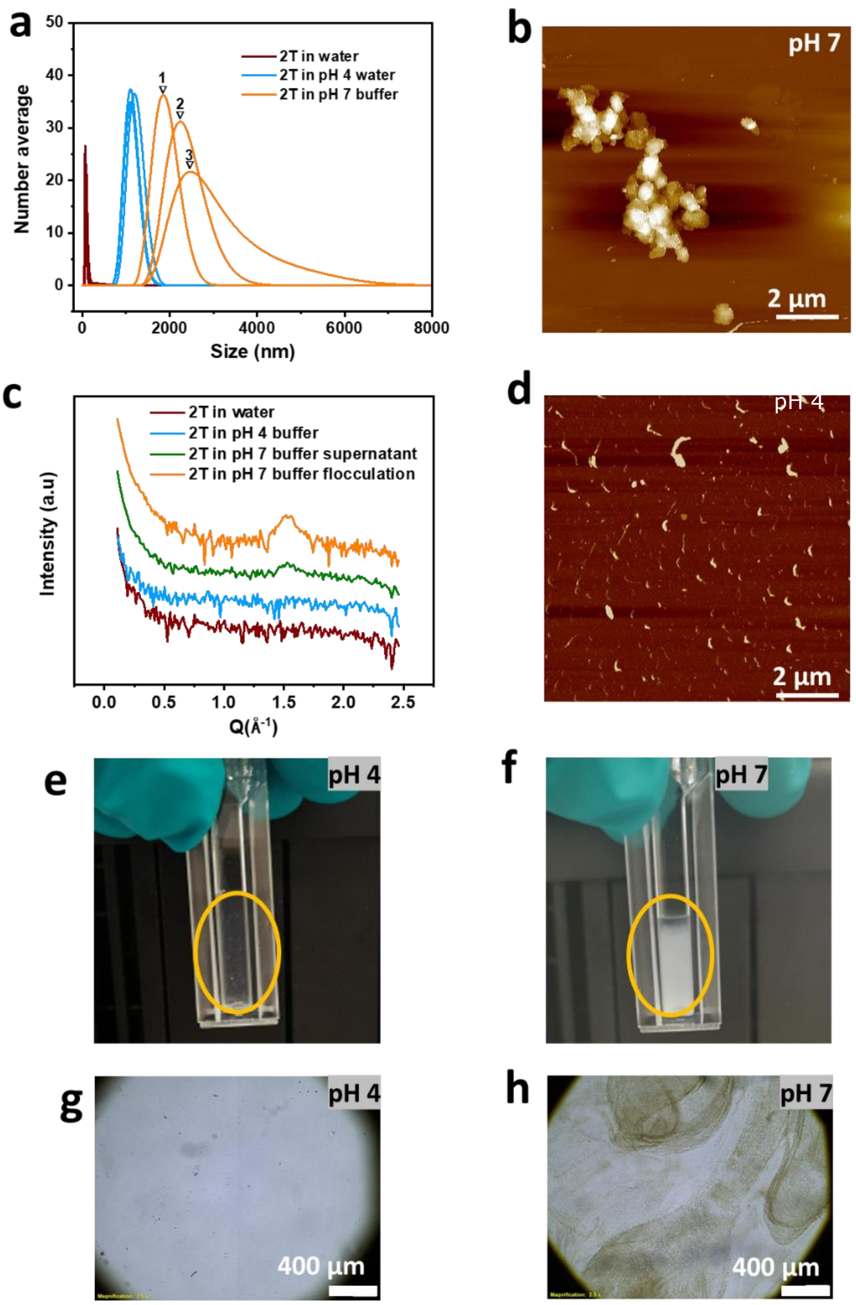

Figure S2. (a) Number average size distributions obtained from dynamic light scattering for 2T solutions in water, in pH 4.0 buffer, and in pH 7.0 buffer. For each three successive distributions are shown. (b) AFM topographic image of 2T after adsorbed on mica from a pH 7.0 buffer solution. (c) SAXS data for 2T solution in water, in pH 4.0 buffer, and in the pH 7.0 buffer supernatant. Data from 2T flocs in pH 7.0 buffer are also shown. Data are shifted vertically for clarity. (d) AFM topographic image of 2T spin-coated from pH 4.0 buffer solution onto a silicon wafer. (e) Photographs of 2T in (f) a pH 4.0 buffer solutions and (g) in a pH 7.0 buffer solution in cuvettes. Optical microscope images of 2T in (f) pH 4.0 buffer and (h) in pH 7.0 buffer solutions.

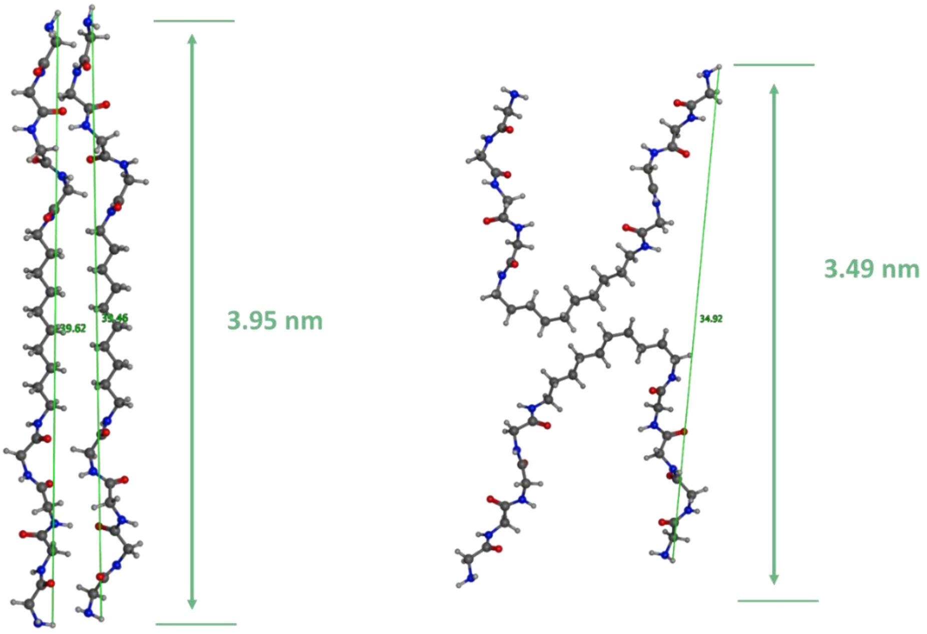

Figure S3. Molecular model of linear aligned $(C_5NGly_4)_2$ molecules (left) and U-shaped aligned $(C_5NGly_4)_2$ molecules (right). The end-to-end lengths are shown for each. Constraints on the dihedral angles (ϕ = –76.9 °, ψ = 145.3°) have been applied.

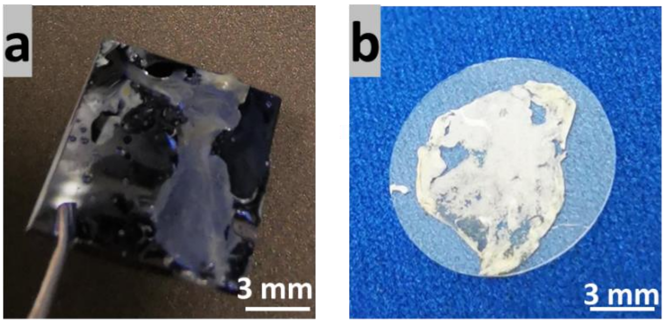

Figure S4. (a) Optical photographs of 2T films freshly deposited onto silicon from the oil/water interface, and (b) deposited on a glass substrate followed by purification with toluene.

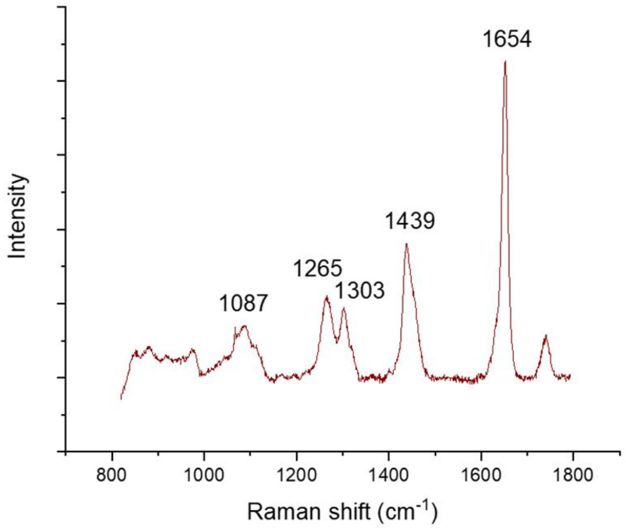

Figure S5. Raman spectrum of pure sunflower oil. The characteristics peaks are identified.

Analysis of the GISAXS data

The GISAXS pattern was further analyzed with the aim to estimate the thickness of the regions composing the lamellar structure.

We recall that the SAXS intensity can be well described by the general equation:

$$I(q) = \Delta\rho^2 P(q) S(q) \quad \text{(S1)}$$

where $\Delta\rho^2$ is the contrast term defined by the electron density difference within the system, $P(q)$ is the so-called form factor, describing the scattered intensity from the objects of a certain shape and size, and $S(q)$ is the structure factor, describing the spatial correlation between adjacent scattering objects.

The many reflections observed in the GISAXS pattern (Figure 4c) are due to the presence of a multilayer structure, generated by the alternating repetition of the peptide and alkyl layers assembled in a stacked fashion (Figure 4d). The absence of the 5th diffraction reflection is directly related to the thickness of the layers. Since the system is molecularly well-defined, destructive interference between the minimum of the lamellar form factor and the 5th reflection maxima occurs. To confirm this, in Figure S6 we present the simulated intensity for the form factor ($P(q)$) of a layer with thickness h = 0.83 nm and the structure factor ($S(q)$) for a multilayered structure with periodicity d = 4.19 nm. For completeness, the simulated background and the total fitted curve are also included in the figure. In these simulations, the scattering from a homogeneous layer with thickness, h, and a lateral size larger than 1000 nm

was used, while the structure factor using paracrystalline disorder was used.[1] The simulations and fitting were performed using MATLAB.

It can be clearly seen that the minimum of the form factor cancels the maximum of the structure factor. Assuming that the system is two phase, the thickness of the second phase must be 4.19 nm - 0.83 nm = 3.36 nm.

We note that the mismatch in the relative peak height is most probably due to the texture of the sample, as the fitted data have been extracted from the vertical cut along the q_z direction of the GISAXS patterns.

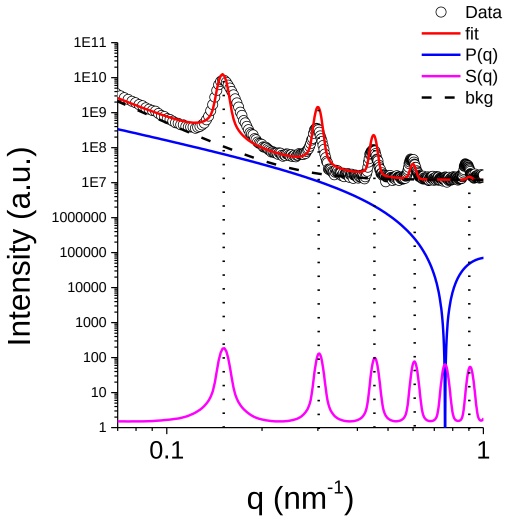

Figure S6. Simulated scattering intensity for the form factor and structure factor for a multi-layered structure.

Pendant Drop Interfacial Tensiometry

A drop shape analyser (Krüss, FTA DSA1000B) was used for pendant drop tensiometry to measure the interfacial tension at the interface between the oil and different aqueous solutions. In these experiments, the aqueous phase was either DI-water, pH 7.0 buffer, 0.5 mg/mL 2T solutions in DI-water, or 0.5 mg/mL 2T in pH 7.0 buffer solution. A drop of the aqueous phase with a volume of approximately 7 µL was injected and hung from a 0.5 mm diameter needle in the less dense oil phase. Images of the drop shape were recorded using the video camera system on the apparatus and analyzed (using FTA32 software) to obtain the interfacial tension value over time. The experiments were conducted in a temperature-controlled room with a temperature of 22 +/-1 °C.

The forces that determine the shape of the pendant drop are mainly the balance of surface tension, buoyancy force and gravitation. The surface tension seeks to minimize the surface area and make the drop take a spherical shape. The difference of gravitation and buoyancy force, on the other hand, stretches the drop from this spherical shape and a typical pear-like shape results. The interfacial tension for a drop with a diameter of D_{eq} at its equator was calculated as:[2]

$$\gamma = \frac{\Delta\rho \cdot g {D_{eq}}^2}{H} \tag{S2}$$

where $\Delta\rho$ is the density difference between water and oil, g is the acceleration due to gravity (taken as 9.8 m/s^2), and H is a shape factor that is decided by the shape of the drop. Here, the water density was taken to be ρ_w = 0.998 g/cm^3 (obtained from the FTA32 software), and the density of sunflower oil[3] was taken as ρ_o = 0.918 g/cm^3. Six replicate measurements were performed on the sunflower oil/DI water interface. The standard deviation was 0.4 mN/m.

In pH 7.0 buffer solutions, there is a self-assembly of 2T platelets. The platelets are large enough to be subject to gravitational effects, which made the interfacial tension measurements unreliable.

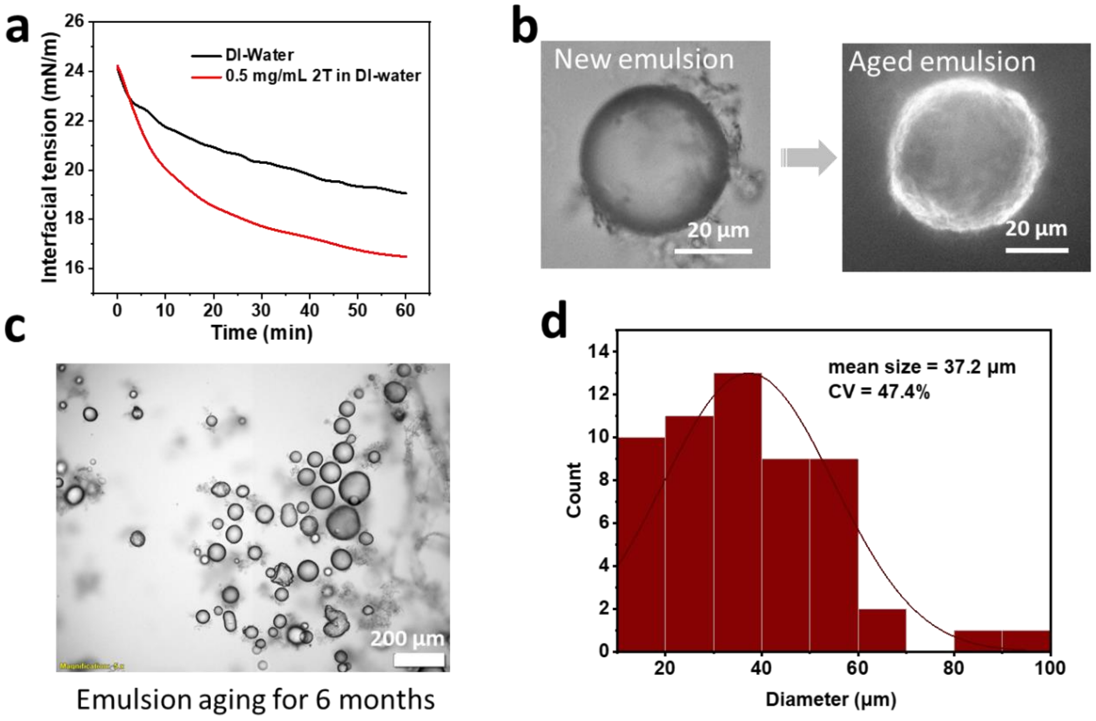

Figure S7. (a) Interfacial tension (from pendant drop tensiometry) for the interface between oil and two different continuous phases (DI water and 2T in DI water) over 60 min. Because of apparent adsorption of impurities in the sunflower oil, the interfacial tension approached an equilibrium value slowly. (b) Optical microscopy images showing oil droplets in water stabilized by 2T after aging for (left) three days and (right) six months. (c) 2T-stabilized oil-in-water emulsion after aging in the dark for six months. (d) Histogram of the 2T-stabilized oil droplet size after aging in the dark for six months.

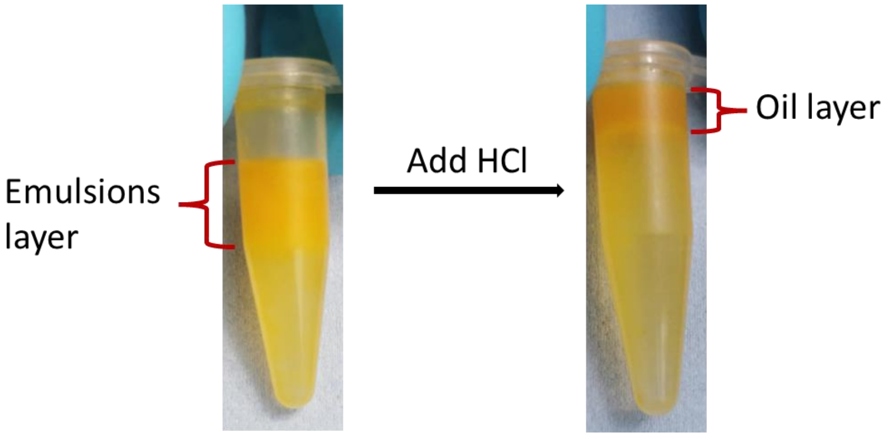

Figure S8. Encapsulation of *β*-carotene and its release after adding 0.1 M HCl.

10% (w/v) *β*-carotene powder was mixed with sunflower oil before emulsification. The *β*-carotene imparted a yellow tint to the oil phase, which was visible under the optical microscope. To trigger the *β*-carotene release, 0.5mL 0.1 M HCl was added to *β*-carotene encapsulated emulsion. Within one minute, a distinct dark yellow oil layer (stained with the *β*-carotene) appeared above the water phase, which provides an illustration that the oil was no longer confined to droplets in the water.

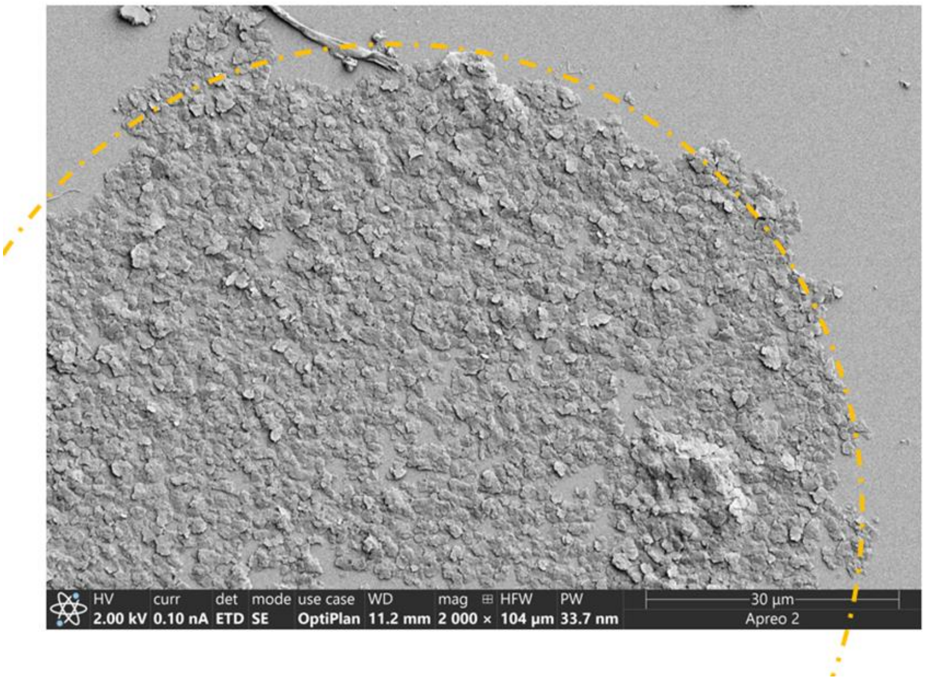

Figure S9. SEM image of a single 2T-stabilized oil drop after being spin-coated and purified by toluene.

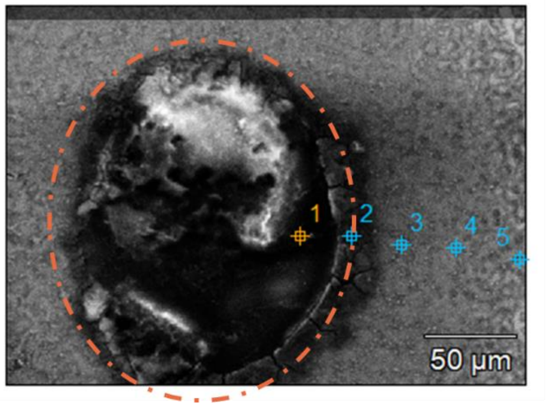

	C-K	O-K	Na-K	P-K
pt1	64.7	5.0	0.1	
pt2	25.7	20.3	14.7	4.1
pt3	4.8	7.7	2.6	0.8
pt4	4.4	4.9	1.5	
pt5	6.4	2.8	1.7	

Figure S10. EDS measurements of the elements (C, O, Na, and P) at five different positions on a dried 2T-stabilized oil drop. An SEM image of the drop is shown at the left.

Video S1. 40-ns coarse-grained simulation showing the formation process of 2T assembly.

Video S2. 10-ns all-atom simulation of 2T attaching to the oil/water interface.

References

(1) Guinier, A.; Fournet, G.; Walker, C. B. *Small-Angle Scattering of X-Rays*; 1955.

(2) Andreas, J. M.; Hauser, E. A.; Tucker, W. B. Boundary Tension by Pendant Drops. *J. Phys. Chem.* **1938**, *42* (7), 1001–1019. https://doi.org/10.1021/j100903a002.

(3) Niă, I.; Neagu, A.; Geacai, S. Study of the Behavior of Some Vegetable Oils during the Thermal Treatment. *Ovidius Univ. Ann. Chem.* **2010**, *21* (1), 5–8.
